# Lymphatic mapping for image-guided radiotherapy in patients with locally advanced uterine cervical cancer: a feasibility study

**DOI:** 10.1186/s13550-023-00989-0

**Published:** 2023-06-12

**Authors:** Judit A. Adam, Edwin Poel, Berthe L. F. van Eck-Smit, Constantijne H. Mom, Lukas J. A. Stalpers, Jaap Stoker, Shandra Bipat

**Affiliations:** 1 Department of Radiology and Nuclear Medicine, Amsterdam UMC Location University of Amsterdam, Meibergdreef 9, 1105AZ Amsterdam, The Netherlands; 2 Department of Gynaecological Oncology, Amsterdam UMC Location University of Amsterdam, Amsterdam, The Netherlands; 3 Center of Gynaecologic Oncology Amsterdam (CGOA), Amsterdam, The Netherlands; 4 Department of Radiotherapy, Amsterdam UMC Location University of Amsterdam, Amsterdam, The Netherlands

**Keywords:** Lymphatic mapping, [^99m^Tc]Tc-nanocolloid, Locally advanced cervical cancer, Radiotherapy

## Abstract

**Background:**

Lymph node metastasis is an important prognostic factor in locally advanced cervical cancer (LACC). No imaging method can successfully detect all (micro)metastases. This may result in (lymph node) recurrence after chemoradiation. We hypothesized that lymphatic mapping could identify nodes at risk and if radiation treatment volumes are adapted based on the lymphatic map, (micro)metastases not shown on imaging could be treated. We investigated the feasibility of lymphatic mapping to image lymph nodes at risk for (micro)metastases in LACC and assessed the radiotherapy dose on the nodes at risk.

**Methods:**

Patients with LACC were included between July 2020 and July 2022. Inclusion criteria were: ≥ 18 years old, intended curative chemoradiotherapy, investigation under anesthesia. Exclusion criteria were: pregnancy and extreme obesity. All patients underwent abdominal MRI, [^18^F]FDG-PET/CT and lymphatic mapping after administration of 6–8 depots of ^99m^Tc]Tc-nanocolloid followed by planar and SPECT/CT images 2–4 and 24 h post-injection.

**Results:**

Seventeen patients participated. In total, 40 nodes at risk were visualized on the lymphatic map in 13/17 patients with a median of two [range 0–7, IQR 0.5–3] nodes per patient, with unilateral drainage in 4/13 and bilateral drainage in 9/13 patients. No complications occurred. The lymphatic map showed more nodes compared to suspicious nodes on MRI or [^18^F]FDG-PET/CT in 8/14 patients. Sixteen patients were treated with radiotherapy with 34 visualized nodes on the lymphatic map. Of these nodes, 20/34 (58.8%) received suboptimal radiotherapy: 7/34 nodes did not receive radiotherapy at all, and 13/34 received external beam radiotherapy (EBRT), but no simultaneous integrated boost (SIB).

**Conclusion:**

Lymphatic mapping is feasible in LACC. Almost 60% of nodes at risk received suboptimal treatment during chemoradiation. As treatment failure could be caused by (micro)metastasis in some of these nodes, including nodes at risk in the radiotherapy treatment volume could improve radiotherapy treatment outcome in LACC.

*Trail registration* The study was first registered at the International Clinical Trial Registry Platform (ICTRP) under number of NL9323 on 4 March 2021. Considering the source platform was not operational anymore, the study was retrospectively registered again on February 27, 2023 at CilicalTrials.gov under number of NCT05746156.

## Introduction

Locally advanced cervical cancer (LACC) [International Federation of Gynaecology and Obstetrics (FIGO) 2018 stage IB3, IIA2-IVA] has a considerable health impact worldwide as cervical cancer accounts for an annual death of approximately 342,000 women [1]. Despite screening and vaccination programs still a substantial number of patients present with locally advanced disease [2].

Curative treatment of women with LACC consists of external beam radiotherapy (EBRT) and simultaneously integrated lymph node boost (SIB) combined with chemotherapy followed by brachytherapy. The presence of suspicious pelvic or para-aortal lymph nodes diagnosed by diagnostic imaging is an important prognostic factor in LACC [2–4]. Therefore, staging by imaging is included in the latest (2018) FIGO staging system for uterine cervical cancer [3, 4]. When available, patients with LACC receive both magnetic resonance imaging (MRI) and 2-deoxy-2-[^18^F]fluoro-D-glucose positron emission tomography computed tomography ([^18^F]FDG-PET/CT), as the accuracies of these imaging modalities differ in the assessment of local invasion and regional lymph node metastases [5, 6]. These imaging modalities are also essential in defining the target volumes for radiotherapy. EBRT is usually limited to the pelvis in the absence of lymph node metastases or when metastasis is limited to a few lymph nodes below the common iliac vessels. EBRT is extended to the para-aortic region, if suspicious nodes are present at or above the level of the common iliac vessels [7]. No imaging method can, however, successfully detect micrometastases. Up to 15% of the patients with LACC have occult para-aortic metastases not shown on imaging [8]. The false negative para-aortic rate on [^18^F]FDG-PET/CT could be up to 24% when pelvic [^18^F]FDG-positive nodes are present [9, 10].

After treatment, local control of the primary tumor is often achieved [11]. Early recurrence of cervical cancer mostly occurs in lymph nodes [12]. For example, in the study of Schmid et al. almost 25% of all patients with recurrent disease presented with recurrence in para-aortic lymph nodes [13]. This suggests that patients with early lymph node recurrence, occult metastases in lymph nodes may initially not have been treated optimally. This may be either due to metastatic lymph nodes which had not been included in the radiation treatment volume or due to insufficient radiation dose on metastatic lymph nodes. Para-aortic surgical staging prior to radiotherapy is considered as a possible solution to eliminate false negative para-aortic lymph nodes on imaging and para-aortic lymphadenectomy is described as optional in international guidelines [14]. Although lymphadenectomy for staging is performed in some institutions [9], it is not a common practice everywhere, as it can delay the start of chemotherapy and can add morbidity, such as lymphedema or infection. Another option is elective para-aortic irradiation in high risk patients [12], but this can cause additional toxicity. Robust patient selection for risk stratification in LACC remains a challenge and several different methods have been suggested. A noninvasive solution to address the high rate of false negative nodes on imaging is preferred to prevent the morbidity of surgery.

**Fig. 1:**
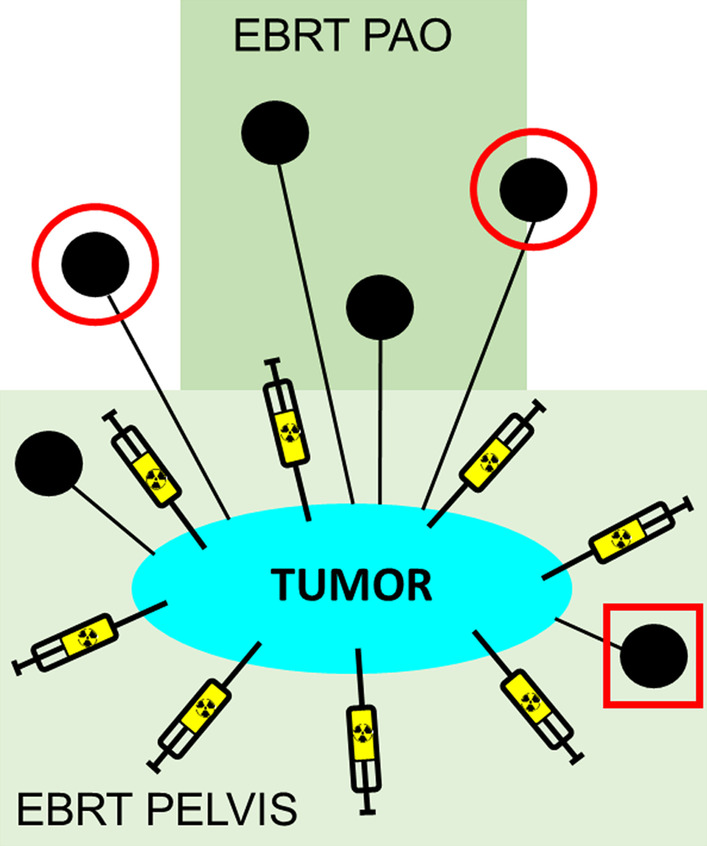
The concept of lymphatic mapping in radiotherapy treatment planning

We hypothesized that lymphatic mapping could address this issue, thereby identifying nodes at risk for metastases. During this procedure, lymph nodes draining from the tumor, not necessarily containing tumor cells, are identified with the aid of a radiopharmaceutical ([^99m^Tc]Tc-nanocolloid) and a gamma camera, much similar to the sentinel node procedure [15]. During lymphatic mapping, an overview of lymph nodes in the surroundings of the tumor can be obtained (= lymphatic map), showing the possible routes of lymph node metastases in the individual patient [16]. All lymph nodes visible on the lymphatic map can be considered as nodes at risk for (micro)metastases (Fig. 1).

After circumferential injection of the radiopharmaceutical, lymph nodes at risk are visualized on the lymphatic map (solid black circles). Some of these nodes are suspicious on imaging (no frame) and full dose radiotherapy, e.g., pelvic and/or para-aortic (PAO) external beam radiotherapy (EBRT, light green rectangles) and simultaneously integrated lymph node boost (SIB) is given to these nodes. Some nodes at risk are only visualized by lymphatic mapping and not treated with radiotherapy at all (red circle) or only receive EBRT, therefore, a suboptimal radiotherapy dose (red square).

If lymphatic mapping in LACC is feasible, radiation treatment volumes can be adapted by including all nodes at risk in the treatment plan. In this case, lymphatic mapping could be used for dose escalation to nodes at risk outside the standard radiation target volume and possibly improve radiotherapy treatment planning and outcome. Additionally, it could be used to constrain the extent and the intensity of the radiation dose in regions without suspicious nodes on imaging or nodes at risk, and thereby limiting toxicity.

In this study, we evaluated lymphatic mapping in LACC. The goals were:

1. To investigate the feasibility of the lymphatic mapping procedure to image lymph nodes at risk for (micro)metastases in women with LACC, and
2. To study the agreement of the lymphatic map with the standard radiotherapy treatment plan. For this objective, we compared the results of the lymphatic map with the routinely used MRI and [^18^F]FDG-PET/CT and retrospectively investigated if all nodes at risk, as identified by lymphatic mapping, received a curative radiation dose.

## Materials and methods

Consecutive patients with LACC at the Amsterdam University Medical Center (UMC) were prescreened for eligibility between July 2020 and July 2022. The inclusion period was set for two years beforehand to ensure timely analysis of the data. Inclusion criteria were: Women ≥ 18 years old, with newly diagnosed, histologically proven LACC (FIGO 2018 stage IB3, IIA2-IVA), planned for curative chemoradiotherapy, who had an investigation under anesthesia (IUA) as part of the standard staging procedure at our institution. Exclusion criteria were: Pregnancy, extreme obesity (body mass index (BMI) > 35; as body posture could hamper circumferential injection of the radiopharmaceutical) and no time slots available for performing the scans.

The study was approved by the medical ethical committee of the Amsterdam UMC, Location University of Amsterdam (NL73563.18.20) and all enrolled patients signed informed consent. The study is registered at the International Clinical Trial Registry Platform (ICTRP) under number of NL9323 and at CilicalTrials.gov under number of NCT05746156.

### Imaging

#### MRI and [^18^F]FDG-PET/CT

All patients underwent abdominal MRI at our institution either at 1.5T (sagittal, coronal and transversal T2-weighted sequences angulated at the cervix, transversal T2 from the kidney to the groins without angulation and transversal DWI [*b*-values 0, 500, 1000]) or at 3T (sagittal T2, transversal T2 bh TE80 of the abdomen, coronal and transversal T2 sequences angulated to the cervix and DWI [*b*-values 100, 500, 1000]) or an MRI with comparable MRI protocol when patients were referred from another institution. After MRI, all patients underwent [^18^F]FDG-PET/CT at our institution after 6 h fasting and administration of 135–300 megabecquerel (MBq), [^18^F]FDG according to BMI, with 135 MBq as reference dosage for a person with 180 cm height and 80 kg weight. PET/CT imaging was combined with diagnostic CT [120 kV, 0.9 pitch and standard Care Dose 4D dose modulation (Siemens Medical Solutions, Erlangen, Germany) with a quality reference tube current of 160 mAs] with administration of intravenous contrast [Ultravist 300 (Bayer Healthcare Pharmaceuticals, Berlin, Germany) 2 ml/kg] and oral water. All patients underwent IUA for staging.

#### Lymphatic mapping

During IUA, the radiopharmaceutical was administered by a gynecologist experienced in sentinel node procedures in cervical cancer as described before [17]. In the Amsterdam UMC, the nuclear medicine physician handles all radioactive material before and after the sentinel node procedure of gynecological cancers and supervises the gynecologist during injection of the radiopharmaceutical, according to local protocols.

Shortly, after viewing the tumor anatomy on MRI in transversal, coronal and sagittal views, palpation of the tumor and adjacent tissue, hemostasis with topical silver nitrate [Bray Group Ltd. Faringdon, England], six to eight depots of 35 MBq [^99m^Tc]Tc-nanocolloid in 0.2 ml each in individual syringes were injected peritumorally with a hypodermic 21G 0.8 × 40 mm needle [BD Microlance™ 3, Becton Dickinson, Fraga (Huesca), Spain]. Preferably eight but at least six injections were evenly distributed around the tumor resulting in a total dose of 210–280 MBq per patient.

Quality control of the injection technique was done as described earlier, including careful preoperative identification of the residual cervical stroma on MRI and visual control of liquid leakage via the cervical canal, into the vagina or otherwise during application by the gynecologist [16]. Complications, such as bleeding or failing administration, were recorded on the clinical research form.

Imaging was done according to our local sentinel node procedure protocol adjusted for lymphatic mapping as follows. Two to four hours after injection of the radiopharmaceutical, 5-min planar imaging including a ^57^Co-floodscource [Rectangular FeatherLite, Eckert&Ziegler, Valencia, Canada] was performed with a 256 × 256 matrix and 1.0 zoom and low energy high-resolution (LEHR)/low-medium-energy (LME) collimator followed by a single-photon emission computed tomography/computed tomography (SPECT/CT) acquisition with a 128 × 128 matrix, 1.0 zoom, 45 × 30 s per views; combined with a low dose CT [30 keV, 40 mAs, 5 mm slice thickness, 1.0 pitch] on a Symbia T16 camera [Siemens Medical Solutions, Erlangen, Germany]. In the first four patients, planar and SPECT/CT images of the abdomen were performed 2–4 h post-injection. Since one of them showed non-visualization of nodes and two of them only unilateral drainage, we hypothesized that more time was necessary for the radiopharmaceutical to reach the lymph nodes. Therefore, the scan protocol was amended in January 2021 and additional planar and SPECT/CT images of the abdomen approximately 24 h after injection (late images) were added to the early images 2–4 h post-injection.

### Image analysis

A nuclear medicine physician with 21 years of experience in sentinel node procedures (JA) read the lymphatic map scans blinded to MRI or [^18^F]FDG-PET/CT imaging. When in doubt, a second nuclear medicine physician (BE) with 37 years of experience was consulted and consensus was reached. A lymph node was considered visualized when the uptake was higher than the surrounding tissue at an anatomically plausible localization, e.g., known lymph node localization. The localizations of the nodes at risk were recorded both on the early and late planar and SPECT/CT images. All visualized nodes were considered nodes at risk.

Nodes were considered suspicious on MRI when a short axis was > 1 cm and/or fulfilled other criteria (ill border, spherical shape and/or diffusion restriction), and on [^18^F]FDG-PET/CT imaging when short axis > 1 cm and/or FDG uptake two times higher than the adjacent vessel.

### Radiotherapy treatment plan

The radiation oncologist determined the radiotherapy treatment volumes according to routine clinical guidelines, based on clinical information (including IUA) and imaging (MRI and [^18^F]FDG-PET/CT), without knowledge of the lymphatic map. The treatment volume for EBRT on the pelvis included the uterus with the primary tumor, vagina (depending of vaginal involvement), both parametria, para-iliac lymph nodes up to the aorta bifurcation to a dose of 45 Gray (Gy) (25 × 1.8 Gy), using an intensity modulated radiotherapy (IMRT) or volumetric modulated arc therapy (VMAT) technique. The target volume was extended to the para-aortic lymph node region up to the renal veins if three or more suspicious pelvic nodes were present or at least one suspicious node was present at or above the level of the common iliac vessels. A simultaneously integrated lymph node boost (SIB) up to 55 in fractions of 2.2 Gy (near the cervical tumor), or 57.5 Gy in fractions of 2.3 Gy (at some distance of the cervical tumor) was delivered to suspicious pelvic lymph nodes; suspicious para-aortic lymph nodes received a SIB of 57.5 Gy in fractions of 2.3 Gy according to EMBRACE II guidelines [12]. EBRT was followed by image-guided brachytherapy with a Fletcher-type applicator (Utrecht, Geneva or Venezia, Nucletron™, Veenendaal, NL), to 36 Gy pulse dose rate (PDR) with 48 pulses of 75 cGy/pulse/hour, preferably to a D90 of 90–94 Gy (EQD2, *α*/*β* = 10 Gy^2^) to the high risk planning target volume (PTV). Radiotherapy was combined with weekly cisplatin 40 mg/m^2^, four courses during EBRT, and a fifth course in the evening prior to brachytherapy.

Optimal radiotherapy was defined as described above. Suboptimal radiotherapy was defined as less than the aimed dose, e.g., if nodes had not been irradiated at all (for example, a non-suspicious para-aortic node on imaging visualized on the lymphatic map) or receiving less than the aimed total dose (for example, a non-suspicious pelvic node on MRI and/or [^18^F]FDG-PET/CT but visualized on the lymphatic map, thereby receiving 45 Gy during EBRT instead of 55–57.5 Gy including SIB).

Suspicious lymph nodes on MRI and [^18^F]FDG-PET/CT were compared to the nodes at risk on the lymphatic map, using the following eleven lymph node areas: left and right para-aortic, left and right common iliac, left and right external iliac, left and right internal iliac, left and right parametrium and presacral. Nodes on the lymphatic map were counted as the node was visualized, either on early or late or both on early and late images. Then, the radiotherapy treatment plan was compared to the lymphatic map regarding the location and total dose received by the nodes at risk. Nodes at risk were divided in three groups. Nodes that received the optimal radiation dose: EBRT and SIB, nodes that received a suboptimal dose: EBRT only and nodes which did not receive any radiation dose at all.

### Data analysis

All continuous variables are expressed as mean ± standard deviation if normally distributed. Nominal data is given in numbers and percentages and non-normally distributed continuous variables are given in median and interquartile range (IQR). Statistical analyses were performed with Rstudio (version 4.2.1).

## Results

### Patients

**Fig. 2:**
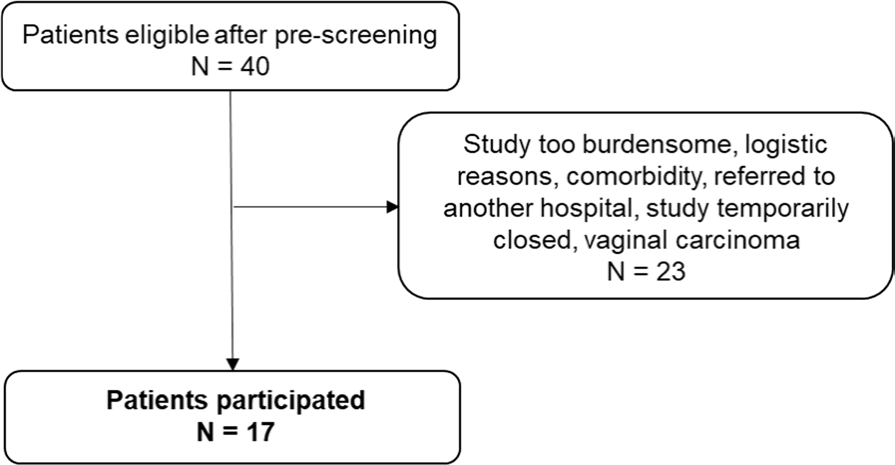
Flowchart showing patient selection for participation

After pre-screening, 40 patients were approached to participate. Eleven patients did not participate as they found participation too burdensome, eight patients could not participate due to logistic reasons, one patient was approached when enrollment was temporarily closed due to processing the amendment, one patient had severe comorbidity, one proved to have a vaginal carcinoma with cervical involvement, and one was referred to another hospital (Fig. 2). Finally, seventeen women participated in the study. Patient and tumor characteristics are shown in Table 1. Out of the 17 included patients 15 had squamous cell carcinoma, and two patients had adenocarcinoma (one poorly differentiated and one mesonephric adenocarcinoma). The mean tumor size was 6.1 cm [SD 1.5 cm].

**Table 1:** Patient and tumor characteristics

*N*	17
**Age (years) (mean [SD])**	49.7 [15.8]
**Histopathology (%)**	
Squamous cell carcinoma	15 (88.2)
Mesonephric adenocarcinoma	1 ( 5.9)
Poorly differentiated adenocarcinoma	1 ( 5.9)
**FIGO 2018 stage (%)**	
IB3	1 (5.9)
IIB	2 (11.8)
IIIC1	12 (70.6)
IIIC2	2 (11.8)
**Tumor size (cm) (mean [SD])**	6.1 [1.5]

Two patients with a BMI > 35 were included in the study, since their body proportions did not hamper successful injection of the radiopharmaceutical according to the screening gynecologist.

### Lymphatic mapping

From the seventeen patients, 16 (94.1%) were injected with eight depots around the tumor, 1/17 patients with six depots due to the anatomical localization of the tumor. None of the patients showed complications due to injection of the radiopharmaceutical and all seventeen included patients underwent imaging. Four patients underwent only early imaging (before the scan protocol was amended) and one patient only late imaging due to postoperative hypotension in the recovery room, hampering transportation to the imaging department.

**Fig. 3:**
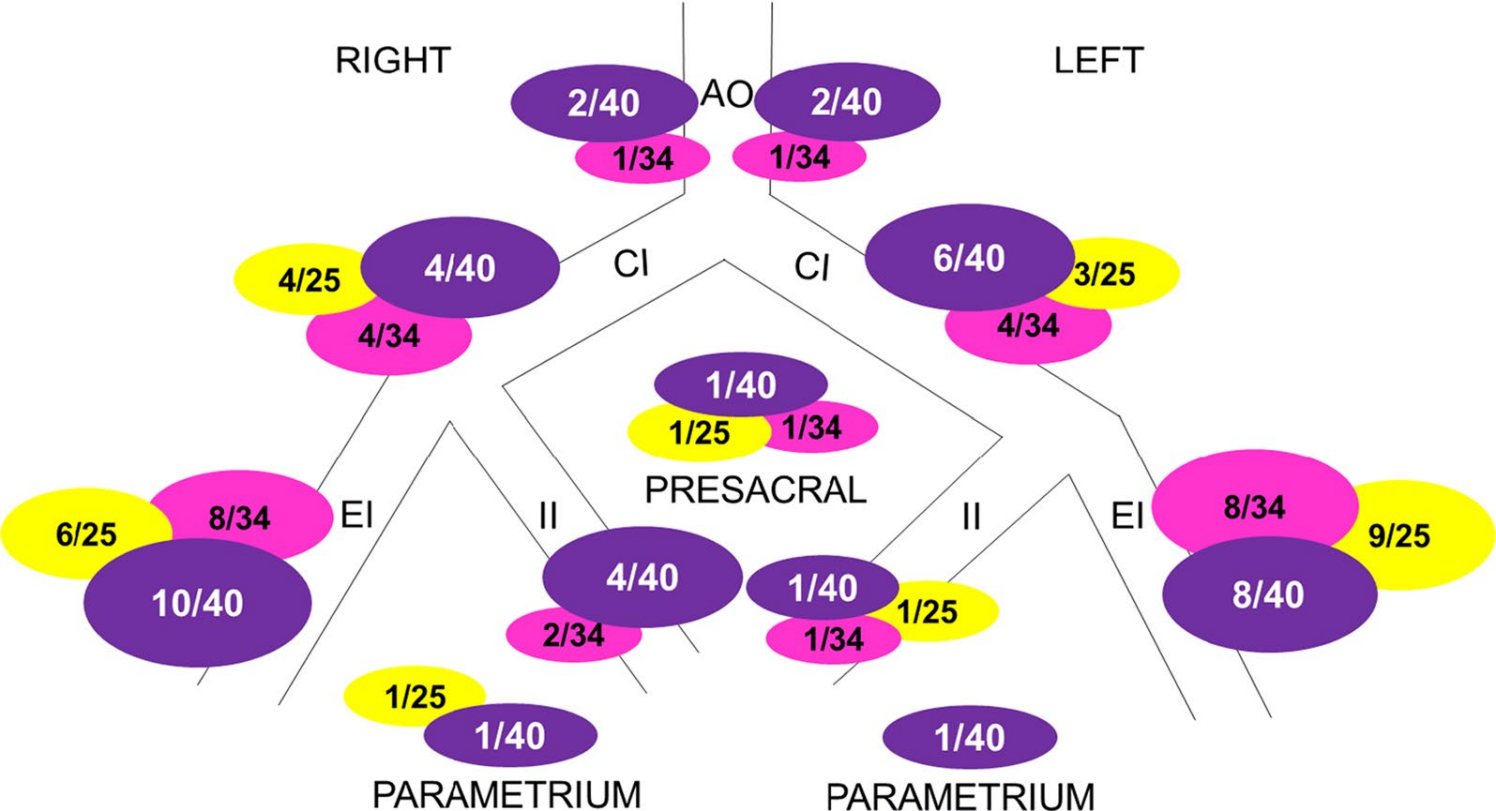
Proportion of visualized lymph nodes on lymphatic map per anatomical localization in the whole population, compared to suspicious nodes on MRI and [^18^F]FDG-PETCT. Anatomical landmarks: aorta (AO), common iliac vessels (CI), external iliac vessels (EI), internal iliac vessels (II), parametrium and presacral. Purple: visualized on lymphatic map, yellow: suspicious on MRI, pink: suspicious on [^18^F]FDG-PET/CT

In total, 40 nodes at risk were visualized on the lymphatic map in seventeen patients, with a median of two [range 0–7, IQR 0.5–3] nodes per patient. In four patients (23.5%), there was no visualization of lymph nodes. Out of the thirteen patients with visualization, the drainage was unilateral in four (30.7%) and bilateral in nine (69.2%) patients. The SPECT/CT showed more nodes than planar imaging in all patients (data not shown). In total, 40 nodes were visualized on SPECT/CT: 19/40 (47.5%) on the left side, 20/40 (50%) on the right side and 1/40 (2.5%) presacral. The anatomical localization of the nodes at risk was as follows: two para-aortic left and two para-aortic right, four common iliac left and six right, ten at the level of the external iliac left and eight right, one at the level of the internal iliac left and four right, one at the parametrium left and one right, and one presacral. The distribution of nodes on the lymphatic map was comparable with the suspicious nodes on MRI and [^18^F]FDG-PETCT with 25 and 34 total suspicious nodes, respectively (Fig. 3).

When looking at visualization on early or late images, in 5/17 patients only one time point of the SPECT/CT was available. In five of the remaining twelve patients (41.7%) the early images showed more nodes than the late images, in two patients (16.7%) the late images showed more nodes, and in the remaining five patients (41.7%) the results between the early and late images were concordant.

#### Comparison to MRI and [^18^F]FDG-PET/CT

When compared to MRI and [^18^F]FDG-PET/CT, suspicious nodes were concordant with the nodes at risk on the lymphatic map in 3/17 (17.6%) of the patients and discordant in 14/17 (82.3%). From the 14 discordant results, in 8/14 (57.1%) patients, the lymphatic map showed more nodes than MRI or [^18^F]FDG-PET/CT, in 6/14 (42.8%) the lymphatic map showed less nodes than the MRI or [^18^F]FDG-PET/CT (Fig. 3).

#### Comparison to radiotherapy treatment plan

From the seventeen patients, sixteen had curative chemoradiotherapy. One patient received radical surgical treatment after the diagnostic phase instead of radiotherapy. In this patient all suspicious lymph nodes on imaging turned out to be tumor negative on pathological examination, therefore radiotherapy was not indicated.

**Fig. 4:**
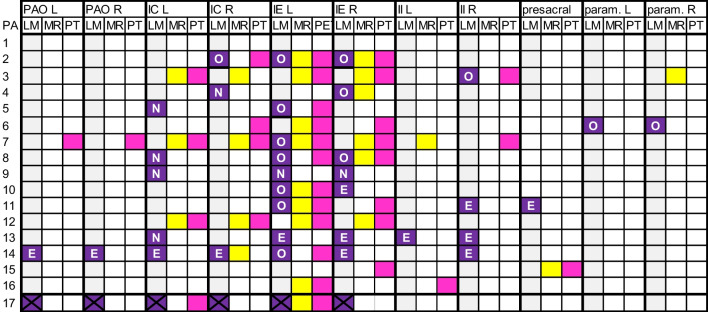
Visualized lymph nodes on lymphatic map (LM; purple), magnetic resonance imaging (MR; yellow) and [^18^F]FDG-PET/CT (PT; pink) per patient (PA; 1–17) and anatomical localization: para-aortic left (PAO L), para-aortic right (PAO R), iliaca communis left (IC L) iliaca communis right (IC R), iliaca externa left (IE L), iliaca externa right (IE R), iliaca interna left (II L), iliaca interna right (II R), presacral, parametrium left (param. L) and parametrium right (param. R). *Abbreviations* O, optimal radiotherapy; E, external beam radiotherapy only, N, no received radiotherapy dose. *Note* No visualization on the lymphatic map in patient number 1, 12, 15 and 16; nodes of patient number 17 were not irradiated as patient was treated surgically

As four patients had non-visualization, 34 lymph nodes were visualized on the lymphatic map in 12/16 patients. Of the 34 nodes, 20 (58.8%) nodes received suboptimal radiotherapy: 7/20 nodes did not receive radiotherapy at all, and 13/20 nodes received EBRT, but no SIB. The remaining 14/34 (41.2%) nodes received optimal radiotherapy, consisting of EBRT and, in case of suspicious nodes on MRI and/or [^18^F]FDG-PET/CT, SIB as well (Fig. 4).

**Fig. 5:**
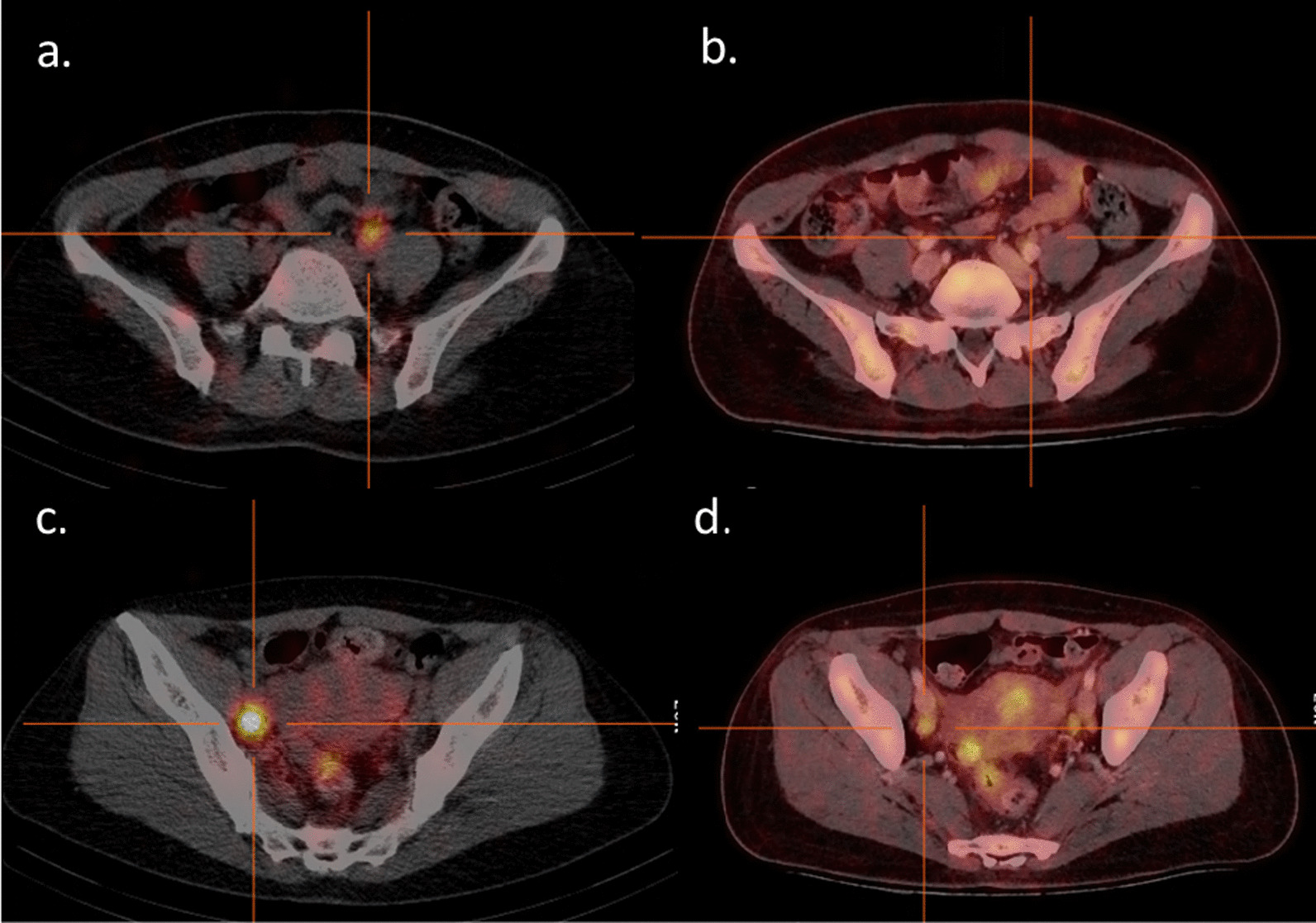
Lymphatic map (fused axial SPECT/CT images 24 h post-injection of [^99m^Tc]Tc-nanocolloid) and [^18^F]FDG-PET/CT images (fused axial images 1 h post-injection of [^18^F]FDG) of a patient with stage rIIIC1 squamous cell carcinoma. Upper row: lymph node at the level of the left common iliac vessel, not irradiated as it was not suspicious on MRI or [^18^F]FDG-PET/CT imaging. **a** Lymphatic map: visualized **b** [^18^F]FDG-PET/CT: no [^18^F]FDG uptake, short axis < 1 cm. Lower row: lymph node at the level of the left external iliac vessel, irradiated as it was suspicious on [^18^F]FDG-PET/CT imaging. **c** Lymphatic map: visualized. **d** [^18^F]FDG-PET/CT: pathological [^18^F]FDG uptake and 1.3 cm short axis

Figure 5 shows an example of an optimally irradiated and not irradiated lymph node on the lymphatic map in a patient with stage rIIIC1 LACC.

## Discussion

To our knowledge, this is the first study showing the feasibility of lymphatic mapping in LACC. Our study shows that lymphatic mapping can safely be executed in patients with LACC. The injection of the radiopharmaceutical was without complications and imaging could take place 2–4 and 24 h after investigation under anesthesia. Based on the results, we conclude that in our setting both early and late imaging is necessary for the highest yield of nodes on the lymphatic map. SPECT/CT should be performed for lymphatic mapping considering that (as expected) SPECT/CT showed more nodes than planar images, and SPECT/CT provides anatomical landmarks for exact lymph node localization which is necessary for treatment planning.

In four patients, there was no visualization of lymph nodes and not every patient showed bilateral drainage of the radiopharmaceutical. We need to take into account that while during sentinel node procedures, it is crucial to harvest the sentinel nodes on both sides of the tumor to gain information on lymph node metastases and avoid lymphadenectomy, in lymphatic mapping the aim is different. During this procedure, the goal is to visualize nodes at risk, e.g., the potential metastatic lymph nodes, including the ones with micrometastases. These nodes could be the ones which are false negative on MRI and [^18^F]FDG-PET/CT and where lymph node recurrence could occur after treatment is completed.

There are no comparable studies in LACC. There is one study of Cibula et al. [16] in which a sentinel node procedure was performed with [^99m^Tc]Tc-nanocolloid and patent blue, without imaging, in 44 patients with cervical cancer FIGO 2009 stages IB1 and IIA ≥ 3 cm, IB2 and selected IIB, who underwent surgical resection. In this study, 23% of the patients did not show any drainage and 41% of the patients unilateral drainage, respectively, comparable to our study. The relative low number of nodes at risk in our study is an unexpected finding and we have no explanation for this. Although the number of nodes at risk in our study is in line with the number of harvested sentinel nodes in the study of Cibula et al., they did not perform preoperative imaging, and therefore, the number of radioactive nodes could be underestimated in their study.

In our study, in 8/14 (57.1%) patients the lymphatic map showed more nodes at risk compared to suspicious nodes on MRI or [^18^F]FDG-PET/CT. When compared to the radiotherapy treatment plan, based on suspicious nodes on MRI and [^18^F]FDG-PET/CT, 20/34 (58.8%) nodes did not receive an optimal radiotherapy dose (e.g., not irradiated or EBRT only) during chemoradiation. Although there is no certainty that the nodes at risk contain tumor metastases, as we did not perform histopathological investigation, it is plausible that some of these nodes at risk could be the false negative ones on MRI and [^18^F]FDG-PET/CT, and responsible for lymph node recurrence. To improve patient outcome, an option could be to additionally irradiate all nodes at risk. As in our study, the number of nodes at risk is limited, when targeted radiotherapy treatment techniques are applied only to lymph nodes at risk (e.g., intensity modulated radiation therapy), additional toxicity could be limited and well defendable in a patient group with a relatively poor prognosis.

Another use of lymphatic mapping could be to guide lymphadenectomy prior to chemoradiation in patients with no suspicious nodes on imaging at the para-aortic level, when only the nodes at risk would be surgically removed instead of a para-aortic lymphadenectomy resulting in less chance of operation related morbidity. Recent studies in prostate cancer show promising results in a comparable approach. In a study by Hinsenveld et al., 100% sensitivity and 94% accuracy for para-aortic nodal staging were shown in patients with intermediate and high risk prostate cancer when sentinel node biopsy was added to PSMA-PET/CT imaging [18]. Another study by de Barros et al. shows improved survival in patients when sentinel node biopsy was added to standard imaging to select pN1 patients with prostate cancer for pelvic radiotherapy [19]. Considering the similarities in the orderly spread of lymph node metastases in prostate and cervical cancer, comparable results could be expected in LACC.

In 6/17 (35.3%) patients the lymphatic map showed less nodes compared to MRI or [18F]FDG-PET/CT. This suggests that the lymphatic map cannot safely be used for radiotherapy dose de-escalation, as potential metastatic nodes could be missed by the lymphatic map and therefore suboptimal treated during curative chemoradiation.

Based on data from the SENTICOL I and SENTICOL II trials, multicenter prospective randomized trials to compare sentinel node biopsy and pelvic lymph node dissection, respectively [20, 21], tumor size ≥ 2 cm was an independent factor of atypical sentinel node localization such as isolated common iliac or para-aortic localization. This could mean that in our cohort with large tumors, more atypical localizations of nodes at risk are present, e.g., in the presacral region.

Tumor size could also influence visualization of nodes on the lymphatic map. However tumor size did not hamper administration of the radiopharmaceutical in our cohort, it is plausible that transport of the radiopharmaceutical is less optimal in larger tumors and more time is necessary for the radiopharmaceutical to reach the lymph nodes due to the changes in the lymphatic bed caused by necrosis. This could mean that the results of the lymphatic map are more robust in a population with relatively smaller LACC.

There are limitations of our study. It has a small sample size, however, customary for a feasibility study. Also, we could not perform early dynamic images (15 min post-injection) as patients were still in the operating room at that time. Theoretically, we could therefore have missed some nodes. However, the suggested time for imaging is 2 h post-injection during the sentinel node procedure [22]. Finally, we did not perform histopathological confirmation of all nodes at risk since it is not always feasible as these nodes are often located at elusive anatomical regions.

For the future, it is important to evaluate whether adding lymphatic mapping to radiotherapy treatment planning results in survival benefit. Whether the use of lymphatic map will have more benefit in patients with or without para-aortic nodes on MRI or [^18^F]FDG-PET/CT still needs to be elucidated.

Therefore, a prospective trial with sufficient length of follow-up should be conducted with overall survival, recurrent-free survival, toxicity and quality of life as endpoints. Our study is a first step toward conducting such a trial, as we have shown that the technique of the lymphatic mapping is safe and feasible in patients with LACC.

## Conclusion

In conclusion, lymphatic mapping is feasible in patients with LACC. During lymphatic mapping, there was visualization of lymph nodes in the majority of patients and there were no procedure related complications. Almost 60% of the nodes at risk received suboptimal treatment during chemoradiation. As treatment failure could be caused by (micro)metastases in these nodes, including nodes at risk in the radiotherapy treatment volume could optimize treatment of LACC. Additional toxicity could be accepted in this patient group with a high chance of recurrent disease and relative poor survival.

The efficacy of lymphatic mapping needs to be assessed in a larger prospective cohort. This feasibility study is an important step toward conducting such a trial.

## Data Availability

The images and other data generated during and/or analyzed during the current study are available from the corresponding author on reasonable request.

## Abbreviations

[^18^F]FDG: 2-Deoxy-2-[^18^F]fluoro-d-glucose
BMI: Body mass index
EBRT: External beam radiotherapy
FIGO: International Federation of Gynaecology and Obstetrics
IQR: Interquartile range
IUA: Investigation under anesthesia
LACC: Locally advanced cervical cancer
MRI: Magnetic resonance imaging
PAO: Para-aortic
PET/CT: Positron emission tomography/computed tomography
SIB: Simultaneous integrated boost
SPECT/CT: Single-photon emission computed tomography/computed tomography

## References

[1] Bray F, Ferlay J, Soerjomataram I, Siegel RL, Torre LA, Jemal A (2018). Global cancer statistics 2018: GLOBOCAN estimates of incidence and mortality worldwide for 36 cancers in 185 countries. CA Cancer J Clin.

[2] Monk BJ, Tan DSP, Hernandez Chagui JD, Takyar J, Paskow MJ, Nunes AT (2022). Proportions and incidence of locally advanced cervical cancer: a global systematic literature review. Int J Gynecol Cancer.

[3] Bhatla N, Berek JS, Cuello Fredes M, Denny LA, Grenman S, Karunaratne K (2019). Revised FIGO staging for carcinoma of the cervix uteri. Int J Gynaecol Obstet.

[4] Corrigendum to "Revised FIGO staging for carcinoma of the cervix uteri" [Int J Gynecol Obstet 145(2019) 129–135]. Int J Gynaecol Obstet. 2019;147:279–80. 10.1002/ijgo.12969. 10.1002/ijgo.12969 31571232

[5] Woo S, Panebianco V, Narumi Y, Del Giudice F, Muglia VF, Takeuchi M (2020). Diagnostic performance of vesical imaging reporting and data system for the prediction of muscle-invasive bladder cancer: a systematic review and meta-analysis. Eur Urol Oncol.

[6] Adam JA, van Diepen PR, Mom CH, Stoker J, van Eck-Smit BLF, Bipat S (2020). [(18)F]FDG-PET or PET/CT in the evaluation of pelvic and para-aortic lymph nodes in patients with locally advanced cervical cancer: a systematic review of the literature. Gynecol Oncol.

[7] Cibula D, Potter R, Planchamp F, Avall-Lundqvist E, Fischerova D, Haie Meder C (2018). The European Society of Gynaecological Oncology/European Society for Radiotherapy and Oncology/European Society of Pathology guidelines for the management of patients with cervical cancer. Int J Gynecol Cancer.

[8] Gouy S, Morice P, Narducci F, Uzan C, Martinez A, Rey A (2013). Prospective multicenter study evaluating the survival of patients with locally advanced cervical cancer undergoing laparoscopic para-aortic lymphadenectomy before chemoradiotherapy in the era of positron emission tomography imaging. J Clin Oncol.

[9] Thelissen AAB, Jurgenliemk-Schulz IM, van der Leij F, Peters M, Gerestein CG, Zweemer RP (2022). Upstaging by para-aortic lymph node dissection in patients with locally advanced cervical cancer: a systematic review and meta-analysis. Gynecol Oncol.

[10] Uzan C, Souadka A, Gouy S, Debaere T, Duclos J, Lumbroso J (2011). Analysis of morbidity and clinical implications of laparoscopic para-aortic lymphadenectomy in a continuous series of 98 patients with advanced-stage cervical cancer and negative PET-CT imaging in the para-aortic area. Oncologist.

[11] Potter R, Haie-Meder C, Van Limbergen E, Barillot I, De Brabandere M, Dimopoulos J (2006). Recommendations from gynaecological (GYN) GEC ESTRO working group (II): concepts and terms in 3D image-based treatment planning in cervix cancer brachytherapy-3D dose volume parameters and aspects of 3D image-based anatomy, radiation physics, radiobiology. Radiother Oncol.

[12] Potter R, Tanderup K, Kirisits C, de Leeuw A, Kirchheiner K, Nout R (2018). The EMBRACE II study: the outcome and prospect of two decades of evolution within the GEC-ESTRO GYN working group and the EMBRACE studies. Clin Transl Radiat Oncol.

[13] Schmid MP, Franckena M, Kirchheiner K, Sturdza A, Georg P, Dorr W (2014). Distant metastasis in patients with cervical cancer after primary radiotherapy with or without chemotherapy and image guided adaptive brachytherapy. Gynecol Oncol.

[14] Cibula D, Potter R, Planchamp F, Avall-Lundqvist E, Fischerova D, Meder CH (2018). The European Society of Gynaecological Oncology/European Society for Radiotherapy and Oncology/European Society of Pathology guidelines for the management of patients with cervical cancer. Radiother Oncol.

[15] Cibula D, Kocian R, Plaikner A, Jarkovsky J, Klat J, Zapardiel I (2020). Sentinel lymph node mapping and intraoperative assessment in a prospective, international, multicentre, observational trial of patients with cervical cancer: the SENTIX trial. Eur J Cancer.

[16] Cibula D, Kuzel D, Slama J, Fischerova D, Dundr P, Freitag P (2009). Sentinel node (SLN) biopsy in the management of locally advanced cervical cancer. Gynecol Oncol.

[17] Cibula D, Dusek J, Jarkovsky J, Dundr P, Querleu D, van der Zee A (2019). A prospective multicenter trial on sentinel lymph node biopsy in patients with early-stage cervical cancer (SENTIX). Int J Gynecol Cancer.

[18] Hinsenveld FJ, Wit EMK, van Leeuwen PJ, Brouwer OR, Donswijk ML, Tillier CN (2020). Prostate-specific membrane antigen PET/CT combined with sentinel node biopsy for primary lymph node staging in prostate cancer. J Nucl Med.

[19] de Barros HA, Duin JJ, Mulder D, van der Noort V, Noordzij MA, Wit EMK (2023). Sentinel node procedure to select clinically localized prostate cancer patients with occult nodal metastases for whole pelvis radiotherapy. Eur Urol Open Sci.

[20] Lecuru F, Mathevet P, Querleu D, Leblanc E, Morice P, Darai E (2011). Bilateral negative sentinel nodes accurately predict absence of lymph node metastasis in early cervical cancer: results of the SENTICOL study. J Clin Oncol.

[21] Mathevet P, Lecuru F, Uzan C, Boutitie F, Magaud L, Guyon F (2021). Sentinel lymph node biopsy and morbidity outcomes in early cervical cancer: results of a multicentre randomised trial (SENTICOL-2). Eur J Cancer.

[22] Giammarile F, Bozkurt MF, Cibula D, Pahisa J, Oyen WJ, Paredes P (2014). The EANM clinical and technical guidelines for lymphoscintigraphy and sentinel node localization in gynaecological cancers. Eur J Nucl Med Mol Imaging.

